# α-synuclein seed amplification assay sensitivity may be associated with cardiac MIBG abnormality among patients with Lewy body disease

**DOI:** 10.1038/s41531-024-00806-y

**Published:** 2024-10-21

**Authors:** Masanori Kurihara, Katsuya Satoh, Ryosuke Shimasaki, Keiko Hatano, Kensuke Ohse, Kenichiro Taira, Ryoko Ihara, Mana Higashihara, Yasushi Nishina, Masashi Kameyama, Atsushi Iwata

**Affiliations:** 1 Department of Neurology, Tokyo Metropolitan Institute for Geriatrics and Gerontology, Tokyo, Japan; 2 Integrated Research Initiative for Living Well with Dementia, Tokyo Metropolitan Institute for Geriatrics and Gerontology, Tokyo, Japan; 3 Department of Health Sciences, Unit of Medical and Dental Sciences, Nagasaki University Graduate School of Biomedical Sciences, Nagasaki, Japan; 4 Research Team for Neuroimaging, Tokyo Metropolitan Institute for Geriatrics and Gerontology, Tokyo, Japan

**Keywords:** Diagnostic markers, Parkinson's disease, Dementia, Movement disorders, Parkinson's disease

## Abstract

Although α-synuclein seed amplification assays (α-syn SAA) are promising, its sensitivity may be affected by heterogeneity among patients with Lewy body disease (LBD). We evaluated whether α-syn SAA sensitivity is affected by patient heterogeneity, using ^123^I-meta-iodobenzylguanidine (MIBG) cardiac scintigraphy in early drug-naïve patients. Thirty-four patients with clinically established or probable Parkinson’s disease (PD) and seven with dementia with Lewy bodies (DLB) or prodromal DLB were included. While 85.2% of patients with abnormal cardiac MIBG were α-syn SAA positive, only 14.3% were positive among those with normal scans. Logistic regression analysis showed that MIBG positivity was the only significant variable associated with α-syn SAA positivity (odds ratio 74.2 [95% confidence interval 6.1–909]). Although α-syn SAA is sensitive for LBD in patients with abnormal MIBG, the sensitivity may be lower in those with normal MIBG. Further studies are necessary to evaluate the association between patient heterogeneity and α-syn SAA sensitivity.

## Introduction

Pathological hallmarks of idiopathic Parkinson’s disease (PD) and dementia with Lewy bodies (DLB) are the presence of Lewy bodies, which consist of aggregated α-synuclein (α-syn). Recent evidence suggests that aggregated α-syn can be detected in the cerebrospinal fluid (CSF) using real-time quaking-induced conversion (RT-QuIC) assays, which was originally developed for prion disease^1^ and later used and validated for Lewy body diseases (LBD)^2–5^. This assay is now often collectively called the α-syn seed amplification assay (SAA). As previous studies have reported high sensitivity even in preclinical phases of LBD, for disorders such as idiopathic REM sleep behavior disorder (iRBD)^6,7^ and pure autonomic failure (PAF)^6^, recently proposed biological classification uses the result of the α-syn SAA to classify whether the patient has LBD^8^ (or recently proposed terminology of neuronal α-synuclein disease^9^). However, the result of a recent large study suggested that the sensitivity of the α-syn SAA may be affected by inter-patient heterogeneity such as the presence or absence of olfactory deficits^10^. Further studies are important to understand the association between the α-syn SAA sensitivity and heterogeneity among patients with LBD.

Recent studies have suggested that there are two different progression pattern of Lewy body pathology^11–15^. It has been proposed that in the body-first subtype of the disease, pathology initiates in the enteric or peripheral autonomic nervous system, including the cardiac sympathetic nerve, and progresses to the dorsal motor nucleus of the vagus in the medulla before ascending through the brainstem. Conversely, in the brain-first subtype, the pathological process is thought to begin in the unilateral olfactory bulb or amygdala, from where it extends to the substantia nigra and subsequently descends through the brainstem^11–15^. Although the former is associated with impaired cardiac sympathetic nerve, symmetric symptoms, and increased frequency of prodromal symptoms (iRBD, autonomic impairment, olfactory deficit) and DLB, the latter is associated with preserved cardiac sympathetic nerve, asymmetric symptoms, and decreased frequency of prodromal symptoms and DLB^11–15^. In the early phase, although further longitudinal studies are needed to support the concept, these subtypes may be identified based on radiological heterogeneity using striatal dopaminergic imaging and ^123^I-meta-iodobenzylguanidine (MIBG) cardiac scintigraphy^12^. However, it remained unknown whether α-syn SAA sensitivity is influenced by this radiological heterogeneity.

Therefore, in this study, we evaluated whether α-syn SAA sensitivity is affected by radiological heterogeneity observed using striatal dopaminergic imaging and cardiac MIBG scintigraphy.

## Results

### Baseline characteristics of the participants

Overall, CSF samples from 105 participants were used for the analysis. Fifty participants received a clinical diagnosis of LBD (PD or DLB). Baseline characteristics did not differ between diagnostic groups (Table 1).

**Table 1:** Baseline characteristics of the participants

	LBD (*n* = 50)		non-LBD (*n* = 55)			
	PD	DLB	MSA	other parkinsonian	AD	Other
n =	42	8	5	14	20	16
Age (years)	72.2 ± 9.8	78.4 ± 3.8	67.8 ± 7.1	72.9 ± 9.1	65.2 ± 11.6	70.8 ± 12.6
Sex (female, %)	45.2%	50.0%	40.0%	64.3%	70.0%	37.5%
LP to SAA (days)	356 ± 89	388 ± 150	368 ± 90	324 ± 66	368 ± 114	360 ± 110

*AD* Alzheimer’s disease, *DLB* dementia with Lewy bodies, *LBD* Lewy body diseases, *LP* lumbar puncture, *MSA* multiple system atrophy, *PD* Parkinson’s disease, *SAA* seed amplification assay.

### Diagnostic performance of α-syn SAA for differentiating LBD versus other diseases

**Fig. 1:**
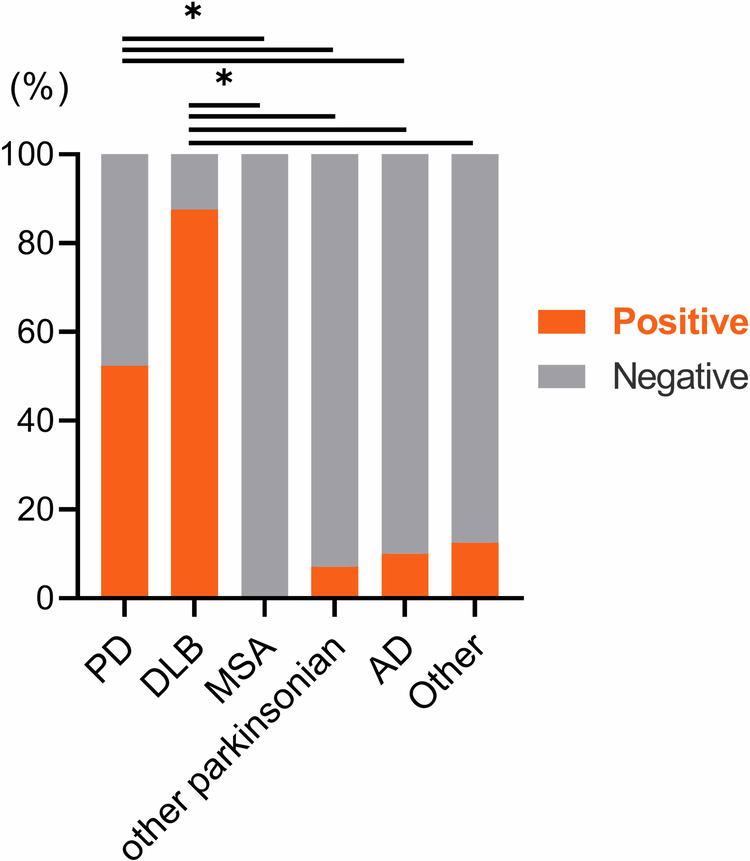
The frequency of α-syn SAA positivity in each diagnostic group. *p < 0.05. AD Alzheimer’s disease, DLB dementia with Lewy bodies, MSA multiple system atrophy, PD Parkinson’s disease.

The positive rates of α-syn SAA for PD and DLB were 52.4% and 87.5%, respectively, which were significantly higher than those in other diagnostic groups (Fig. 1). Example SAA curves and interpretations are provided in Supplementary Fig. 2. The diagnostic performances of α-syn SAA in several situations are summarized in Table 2. While the specificity of α-syn SAA for the diagnosis of LBD was high (91%) and the sensitivity for DLB was also high (88%), the sensitivity for PD was 52%, which was lower than expected.

**Table 2:** Diagnostic performance of α-syn SAA in several situations

	Sensitivity	Specificity	Accuracy	Positive likelihood ratio	Negative likelihood ratio
LBD vs non-LBD (*n* = 105)	58% (43–72)	91% (80–97)	75% (66–83)	6.4 (2.7–15.2)	0.46 (0.33–0.65)
PD vs other Parkinsonian (including MSA) (*n* = 61)	52% (36–68)	95% (75–100)	66% (53–78)	10.5 (1.5–72.3)	0.50 (0.36–0.70)
DLB vs AD (*n* = 28)	88% (47–100)	90% (67–99)	89% (71–98)	8.3 (2.2–31.6)	0.14 (0.02–0.88)

*AD* Alzheimer’s disease, *DLB* dementia with Lewy bodies, *LBD* Lewy body diseases (PD + DLB), *MSA* multiple system atrophy, *PD* Parkinson’s disease.

### Radiological heterogeneity among participants with LBD and association between α-syn SAA positivity and cardiac MIBG abnormality

**Fig. 2:**
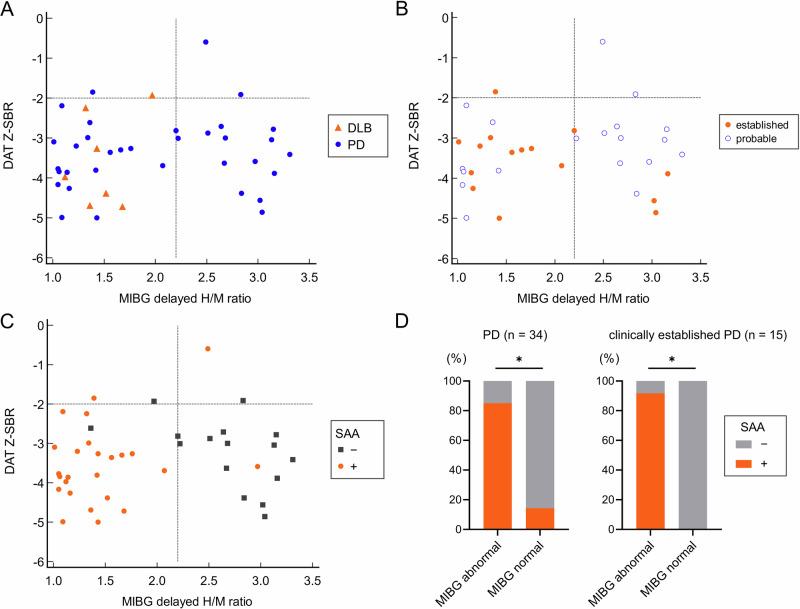
Radiological heterogeneity and α-syn SAA positivity in patients with LBD. **A**–**C** Striatal DAT Z-SBR and cardiac MIBG delayed H/M ratio of each patient. Vertical and horizontal dot lines represent predetermined cut-off lines. **A** Although normal cardiac MIBG was observed in 41% (14/34) of patients with PD, all patients with DLB showed decreased cardiac MIBG H/M ratio irrespective of DAT measure. **B** Plots are limited to patients with PD. Not only those meeting the probable PD criteria but also 15% of those meeting clinically established MDS-PD criteria showed normal cardiac MIBG results. **C** Plots of all patients with LBD and their α-syn SAA positivity. **D** The difference in α-syn SAA positivity between MIBG normal and abnormal groups was significant for both analyses limited to patients with PD (85.0% vs 14.3%, *p* < 0.001) and patients with clinically established PD (91.7% vs 0%, *p* < 0.01).

Of the 50 patients with a clinical diagnosis of LBD, 45 had available results for both DAT SPECT and MIBG cardiac scintigraphy (the patients with missing results were excluded). Furthermore, we excluded four patients with a diagnosis of PD not fulfilling the MDS-PD clinically established or probable criteria. The remaining 41 patients were included in further analysis. Thirty-four patients were in the PD group and seven were in the DLB group. None were suspected to have familial forms and genetic testing was not conducted. Twenty-seven were evaluated within two years from disease onset. The SAA positivity was numerically lower in those evaluated within 2 years from disease onset but the difference was nonsignificant (56% vs 71%, *p* = 0.50). The indices of striatal DAT and cardiac MIBG of each patient are plotted in Fig. 2 in reference to a previous study reporting radiological heterogeneity^12^. When displayed in a scatter plot, a wide distribution for both DAT and MIBG measures was observed. Correlation between DAT Z-SBR and MIBG delayed H/M ratio was weak (*r* = 0.12) and nonsignificant (*p* = 0.45). While normal cardiac MIBG was observed in 41% (14/34) of patients with PD, all patients with DLB showed abnormal cardiac MIBG (Fig. 2A). Not only those meeting the probable criteria but also 15% of those meeting clinically established MDS-PD criteria^16^ showed normal cardiac MIBG results (Fig. 2B).

LBD patients with or without MIBG abnormality were similar in age (73.6 ± 10.0 vs. 74.4 ± 6.6), sex (female 48.1% vs 35.7%), and disease duration (1 [1–2.0] vs 1 [1–2.5] years) (Table 3). Although other baseline characteristics were also similar except for the frequency of DLB and geriatric depression scale (Table 3), the frequency of α-syn SAA positivity was significantly higher in patients with MIBG abnormality (85.2% vs 14.3%, *p* < 0.001) (Fig. 2C). This difference remained significant when limiting the analysis to patients with PD or to patients with clinically-established PD (Fig. 2D).

**Table 3:** Baseline characteristics of participants with LBD and the frequency of α-syn SAA positivity grouped by MIBG abnormality

	MIBG abnormal	MIBG normal	*p* value	
*n*	27	14		
Age (years)	73.6 ± 10.0	74.4 ± 6.6	0.78^a^	
Sex (female)	48.1%	35.7%	0.52^b^	
Disease duration (years)	1 [1–2.0]	1 [1–2.5]	0.96^c^	
UPDRS Part 3 score	28.1 ± 15.5	22.8 ± 12.0	0.31^a^	
MMSE	27 [22.5–29]	27 [24.25–29]	0.60	
GDS	5 [3–6.5]	1 [1–4.0]	< 0.01^c^	**
DAT Z-SBR	−3.49 ± 0.89	−3.23 ± 1.10	0.42^a^	
DLB	25.9%	0%	0.075^b^	
LP to SAA (days)	338 ± 88	378 ± 87	0.17^a^	
α-syn SAA positive	85.2%	14.3%	< 0.001^b^	***

*DAT* dopamine transporter, *DLB* dementia with Lewy bodies, *GDS* geriatric depression scale, *LP* lumbar puncture, *MMSE* Mini-Mental State Examination, *SAA* seed amplification assay, *UPDRS* unified Parkinson’s disease rating scale, *Z-SBR* Z-score of the average striatal SBR, **p value < 0.01, ***p value < 0.001.

^a^Student’s t-test.

^b^Fisher’s exact test.

^c^Mann–Whitney *U* test.

When patients were grouped by α-syn SAA positivity, there were no significant differences in baseline characteristics such as age, sex, disease duration, UPDRS part 3 score, and DAT Z-SBR, and disease type among the groups (Supplementary Table 1). However, the prevalence of MIBG abnormalities was significantly higher in patients with a positive α-syn SAA. Furthermore, all MIBG measures, including the early heart-to-mediastinum (H/M) ratio, delayed H/M ratio, and washout rate, were significantly altered and abnormal in this group (Supplementary Table 1). Logistic regression analysis showed that α-syn SAA positivity was significantly associated with MIBG positivity (odds ratio 74.2 [95% confidence interval = 6.1–909]), but not with age, sex, disease duration, and PD or DLB.

## Discussion

**Fig. 3:**
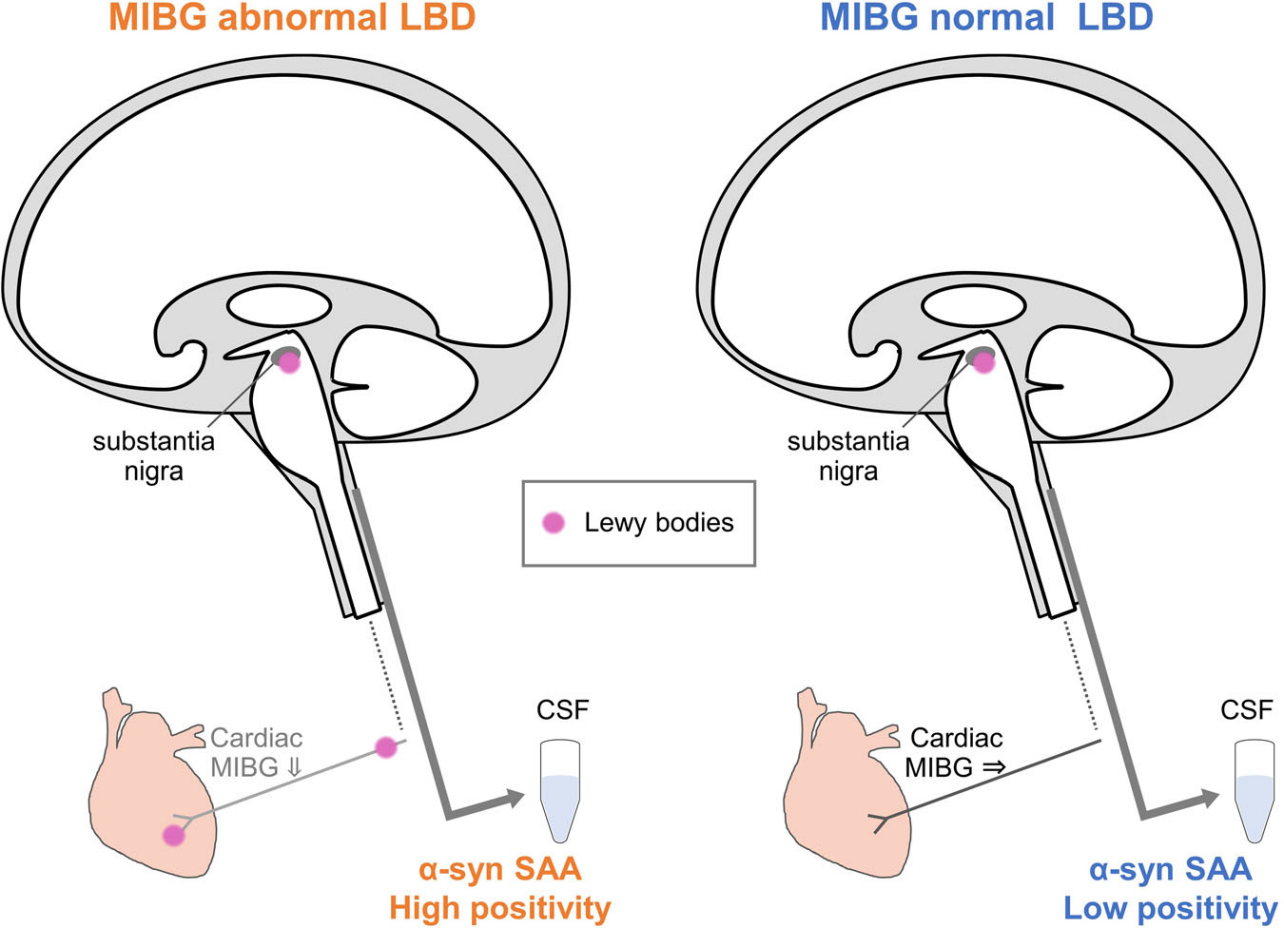
Schematic illustration of summary findings of this study. α-syn SAA positivity was high in patients with MIBG abnormal LBD. From previous reports, these patients should have Lewy bodies at least in the cardiac sympathetic system and substantia nigra in PD. However, α-syn SAA positivity was low in patients with MIBG normal PD. Although neuropathological data is unavailable at this point, from previous reports, most of the patients with non-young-onset and non-familial PD should have Lewy bodies at least in the substantia nigra. (The elements of the figure were created by the author using Adobe Illustrator and Microsoft PowerPoint).

In this study, we confirmed that the RT-QuIC assay we used as α-syn SAA is specific for LBD. Furthermore, our results suggest that the α-syn SAA sensitivity may be affected by inter-patient radiological heterogeneity, namely the presence or absence of cardiac MIBG abnormalities (Fig. 3).

While our study showed high specificity (91%) of α-syn SAA for LBD in line with previous reports, the sensitivity was lower (58%) than that reported in previous studies^5–7,17–31^. This difference could be due to a number of factors including differences in sample handling, assay characteristics, or clinical characteristics of the participants. As a previous study on a different PD cohort using the same α-syn RT-QuIC assay showed a higher sensitivity (unpublished) and a recent large study suggested that the positivity of α-syn SAA may be affected by heterogeneity among participants^10^, we evaluated whether α-syn SAA sensitivity was affected by heterogeneity among participants in our cohort.

MIBG cardiac scintigraphy abnormalities reflect the degeneration of postganglionic cardiac sympathetic nerve terminal^32–34^. Overall, cardiac MIBG uptake is reduced in patients with clinical diagnosis of PD and DLB and the sensitivity is reported to range between 64.5–100% for PD^35^ and 82.4–100% for DLB^36^. In the PD group, up to 40% of patients may show normal cardiac MIBG at the initial scan and a decreased cardiac MIBG at the follow-up^37,38^. This change between the initial scan and the follow-up might be associated with different clinical characteristics^37^. In an autopsy validation study conducted at our institution, although the MIBG H/M ratio strongly correlated with residual cardiac sympathetic fibers, the sensitivity for the LBD pathology was 80%^33^. Those with normal cardiac MIBG and LBD pathology at autopsy (false-negative) showed preserved cardiac sympathetic fibers even at autopsy; thus, suggesting a different progression pattern^33^. Recent reports proposed that the MIBG cardiac scintigraphy is useful for differentiating subtypes of disease progression associated with clinical characteristics^11–15^, the presence of which was also suggested^13,14^ using autopsy data^39^. In this study, because the MIBG cardiac scintigraphy results were available in most participants and were normal in one-third of the patients with LBD, we decided to evaluate whether α-syn SAA sensitivity was associated with cardiac MIBG abnormality. Although α-syn SAA sensitivity in patients with abnormal cardiac MIBG was high (85%) and similar to that observed in previous reports, the sensitivity was as low as 14% in patients with normal cardiac MIBG. The difference was significant and logistic regression analysis confirmed that the association was not confounded by other factors. These results suggest that α-syn SAA sensitivity is affected by the presence of cardiac MIBG abnormality, and that the high rate of MIBG normality in our cohort contributed to the lower sensitivity compared to previous studies.

The difference in pathological distribution and load could be contributing to the difference in α-syn SAA sensitivity (Supplementary Fig. 3). In previous autopsy validation studies, while α-syn SAA was 100% positive in individuals with widespread cortical distribution of Lewy bodies, the sensitivity was lower in those with pathology limited to the amygdala or brainstem^31,40^. As the brain-first LBD is considered to start in amygdala and spread by descending into the brainstem, it is associated with normal cardiac MIBG^11–15^. In this study, patients with normal cardiac MIBG may have had Lewy body pathology relatively limited to the amygdala or brainstem, and thus showed negative α-syn SAA results. Alternatively, body-first LBD is associated with abnormal cardiac MIBG results and the pathology may also persist within the brainstem. Faster clinical progression and accelerated dementia^14,15^ suggest that the pathology may have already spread to the neocortex by the time motor symptoms manifest. Consequently, this subtype often exhibits positive α-syn SAA results (Supplementary Fig. 3). Another possibility may include different α-syn strains (Supplementary Fig. 4) or inhibitory matrix in the CSF of LBD with normal cardiac MIBG^41^. Although recently proposed biological classifications rely on the assumption that α-syn SAA sensitivity is very high from the prodromal phase^8,9^ based on previous studies in iRBD^6,7^ and PAF^6^, these prodromal symptoms may be characteristic only for LBD with abnormal cardiac MIBG^11–15^. Following these results, future studies may be necessary to evaluate whether high α-syn SAA sensitivity can be replicated in the early or prodromal phase of LBD with normal cardiac MIBG.

This study had several limitations. First, diagnoses were clinical, and neuropathological confirmation was unavailable, thus, some of the patients with normal MIBG results may later develop other Parkinsonian disorders. However, diagnoses were made by board-certified neurologists, and analysis regarding radiological heterogeneity was limited to those meeting clinically established or probable PD criteria (MDS criteria), the diagnostic accuracy of which is reported to be 92.5% even for probable PD^42^. Second, all participants were Japanese and genetic testing to rule out genetic forms of LBD was not conducted. Third, we only used our α-syn RT-QuIC assay and a single cut-off to determine α-syn SAA positivity. Using different assays and cut-offs may yield different sensitivities and may show higher sensitivity, especially in the MIBG normal group. Future studies using different α-syn SAA assays in our MIBG normal group samples would be important to further understand the difference in diagnostic characteristics between different assays. Fourth, not all clinical information was blinded to the tester. However, since we used objective criteria to determine SAA positivity and the tester was blinded to the final clinical diagnosis and the results of cardiac MIBG scintigraphy, the main findings of the study are likely not biased by this point. Fifth, although we focused on DAT SPECT and MIBG cardiac scintigraphy results that were available for most of the participants in the biobank, data regarding other important factors associated with heterogeneity among patients with LBD such as the presence of RBD and olfactory function were insufficient to conduct a formal analysis in the present study. We are collecting these data for further studies.

In conclusion, although α-syn SAA is specific for LBD and is also sensitive in patients with MIBG abnormality, the sensitivity may be lower in those with normal cardiac MIBG. Further studies are warranted to evaluate the association between patient heterogeneity and α-syn SAA sensitivity.

## Materials and methods

### Participants

Patients who underwent lumbar puncture for CSF biomarker measurement at the Tokyo Metropolitan Institute for Geriatrics and Gerontology between December 2021 and June 2023 were recruited for the Tokyo Medical Biobank. Most patients were recruited during the initial diagnostic workup and were in the early course of the disease as well as drug-naïve. Those who consented to biobanking with sufficient remaining CSF samples were included in this study. This study was performed in accordance with the tenets of the Declaration of Helsinki and was approved by the Institutional Review Board of the Tokyo Metropolitan Institute for Geriatrics and Gerontology (R23-031). The remaining CSF, serum, and plasma samples were stored as part of the Tokyo Medical Biobank for future study. The clinical diagnosis of each patient was based on the latest information obtained on April 1, 2024. Patients with a diagnosis of PD were considered the PD group and evaluated using the MDS clinical diagnostic criteria^16^. Patients with DLB or prodromal DLB were evaluated using previous criteria^43,44^ and considered the DLB group. Patients with multiple system atrophy (MSA), corticobasal syndrome and progressive supranuclear palsy syndrome, and Alzheimer’s disease (AD) were separated into the MSA, other Parkinsonian disease, and AD groups. CSF biomarker measurement methods and cut-offs for the AD diagnosis were as previously described^45–47^. All patients with other diagnoses including non-AD mild cognitive impairment or dementia, primary progressive aphasia, and amyotrophic lateral sclerosis were enrolled in the Other group. The LBD groups include the patients in the PD and DLB groups.

### CSF sample collection

CSF samples were collected and stored as previously reported with minor modifications. Briefly, CSF samples were obtained using a standard lumbar puncture procedure and the first tube was sent for cell counting and routine biochemical testing. Subsequent CSF samples were directly collected in polypropylene low-binding tubes and centrifuged at 800 × *g* for 10 min at 4 °C. Lastly, 0.5 mL of each sample was aliquoted into 1.5 mL tubes (Proteosave SS 1.5 mL, Sumitomo Bakelite Co., Ltd., Tokyo, Japan) and stored at −80 °C.

### α-syn SAA (RT-QuIC)

The α-syn recombinant protein was expressed and purified as previously described^3^. The RT-QuIC reaction mix consisted of 50 mM PIPES (pH 7.0), 170 mM NaCl, 10 µM thioflavin-T (ThT), 0.1 mM ethylenediaminetetraacetic acid tetrasodium salt hydrate (EDTA), 0.015‰ sodium dodecyl sulfate (SDS), and 0.1 mg/mL human recombinant α-syn protein. Each well of a black 96-well plate with a clear bottom (Nunc 96 well; Sigma-Aldrich, St. Louis, MO, USA) contained 100 μL with two 0.5 mm zirconium/silica beads. Reactions were seeded with 20 μL of undiluted CSF to a final reaction volume of 100 μL. The plates were sealed with a plate sealer film and incubated in a BMG OPTIMA Fluo STAR plate reader at 53 °C for 120 h with intermittent shaking cycles: double orbital with 1 min shake (400 rpm), 15 min rest. ThT fluorescence measurements (450 nm excitation and 480 nm emission) were taken every 15 min. Each sample was run in triplicate, allowing two negative control samples (reactions seeded with prion disease brain homogenate), one positive control (reaction seeded with LBD brain homogenate), an unseeded reaction, and 20 CSF samples to be tested on one plate. The α-syn amyloid formation was monitored for 72 h. The specificity of this assay was confirmed using samples from disease controls such as Creutzfeldt–Jakob disease (Supplementary Fig. 1). Age, sex, initial clinical diagnosis, and date of CSF obtainment were provided, and other detailed clinical information including the final clinical diagnosis and the results of cardiac MIBG scintigraphy were blinded to the tester. A positive reaction is indicated when the ThT fluorescence value exceeds 20,000 arbitrary units in two or more wells within 200 cycles, as per our established protocol. In instances where only a single well of the sample exhibits an elevated value, we take the precautionary measure of retesting the sample. Subsequently, a positive reaction is confirmed if more than one well demonstrates a ThT fluorescence value of 20,000 arbitrary units or greater.

### Radiological assessment

Dopamine transporter (DAT) single photon emission computed tomography (SPECT) images were acquired and evaluated as previously described^48^^,49^. Briefly, 185 MBq of ^123^I-ioflupane (DaT SCAN®, Nihon Medi-Physics, Tokyo, Japan) was administered and images were acquired after 3 h. The specific binding ratios (SBR) of striatal DAT binding were semi-quantitatively calculated using the DAT VIEW software (Nihon Medi-Physics) with the Southampton method^50^ after phantom calibration. The z-score of the average striatal DAT SBR (Z-SBR) for age- and sex-matched Japanese participants^51^ was calculated and a cut-off was set at -2.

^123^I-MIBG cardiac scintigraphy images were acquired and assessed as previously described using smart MIBG (PDRadiopharma Inc., Tokyo, Japan)^33^^,49^. Briefly, 111 MBq of ^123^I-MIBG (PDRadiopharma Inc.) was administered, whereas early and delayed images were obtained after 15–30 min and 3–4 h, respectively. The heart-to-mediastinum (H/M) ratios underwent standardized conversion to a value comparable to that of a medium-energy-type collimator^52^, and washout rates were calculated from early and delayed images^33^. The cut-off for the delayed H/M ratio was set at 2.2.

Abnormalities of DAT SPECT and MIBG cardiac scintigraphy were identified by an expert nuclear medicine physician (M. Kameyama) with reference to these quantitative indices.

### Statistical methods

Statistical analyses were conducted using GraphPad Prism version 9 (GraphPad Software, San Diego, CA, USA), MedCalc Statistical Software version 20.218 (MedCalc Software Ltd., Ostend, Belgium; https://www.medcalc.org; 2023), or R version 4.0.3 (R Foundation for Statistical Computing, Vienna, Austria) and a graphical interface EZR (Saitama Medical Center, Jichi Medical University, Saitama, Japan)^53^. Missing data were handled using a pairwise deletion approach. Categorical variables are expressed as percentages and differences between groups were evaluated using Fisher’s exact test. Pairwise comparisons were performed using Holm’s method. Normally distributed continuous variables are expressed as mean ± standard deviation and differences between groups were tested using the Student’s *t*-test. Continuous variables without a normal distribution are expressed as median (interquartile range) and differences between groups were tested using the Mann–Whitney U test. Correlation was evaluated using Spearman’s method. Logistic regression analysis was performed with α-syn SAA positivity as the dependent variable and age, sex, disease duration, PD or DLB, and MIBG positivity as independent variables. *P* < 0.05 were considered statistically significant.

## Supplementary information

Supplementary Material

## Data Availability

The data supporting the findings of this study are available from the corresponding author upon reasonable request.

## References

[1] Atarashi, R. et al. Ultrasensitive human prion detection in cerebrospinal fluid by real-time quaking-induced conversion. *Nat. Med.* **17**, 175–178 (2011). 21278748 10.1038/nm.2294

[2] Fairfoul, G. et al. Alpha-synuclein RT-QuIC in the CSF of patients with alpha-synucleinopathies. *Ann. Clin. Transl. Neurol.* **3**, 812–818 (2016). 27752516 10.1002/acn3.338 PMC5048391

[3] Sano, K. et al. Prion-Like Seeding of Misfolded alpha-Synuclein in the Brains of Dementia with Lewy Body Patients in RT-QUIC. *Mol. Neurobiol.* **55**, 3916–3930 (2018). 28550528 10.1007/s12035-017-0624-1 PMC5884914

[4] Nakagaki, T., Nishida, N. & Satoh, K. Development of alpha-synuclein real-time quaking-induced conversion as a diagnostic method for alpha-synucleinopathies. *Front Aging Neurosci.* **13**, 703984 (2021). 34650422 10.3389/fnagi.2021.703984 PMC8510559

[5] Grossauer, A. et al. alpha-synuclein seed amplification assays in the diagnosis of synucleinopathies using cerebrospinal fluid-A systematic review and meta-analysis. *Mov. Disord. Clin. Pract.* **10**, 737–747 (2023). 37205253 10.1002/mdc3.13710 PMC10187020

[6] Rossi, M. et al. Ultrasensitive RT-QuIC assay with high sensitivity and specificity for Lewy body-associated synucleinopathies. *Acta Neuropathol.* **140**, 49–62 (2020). 32342188 10.1007/s00401-020-02160-8 PMC7299922

[7] Iranzo, A. et al. Detection of alpha-synuclein in CSF by RT-QuIC in patients with isolated rapid-eye-movement sleep behaviour disorder: a longitudinal observational study. *Lancet Neurol.* **20**, 203–212 (2021). 33609478 10.1016/S1474-4422(20)30449-X

[8] Hoglinger, G. U. et al. A biological classification of Parkinson’s disease: the SynNeurGe research diagnostic criteria. *Lancet Neurol.* **23**, 191–204 (2024). 38267191 10.1016/S1474-4422(23)00404-0

[9] Simuni, T. et al. A biological definition of neuronal alpha-synuclein disease: towards an integrated staging system for research. *Lancet Neurol.* **23**, 178–190 (2024). 38267190 10.1016/S1474-4422(23)00405-2

[10] Siderowf, A. et al. Assessment of heterogeneity among participants in the Parkinson’s Progression Markers Initiative cohort using alpha-synuclein seed amplification: a cross-sectional study. *Lancet Neurol.* **22**, 407–417 (2023). 37059509 10.1016/S1474-4422(23)00109-6 PMC10627170

[11] Borghammer, P. & Van Den Berge, N. Brain-first versus gut-first Parkinson’s disease: a hypothesis. *J. Parkinsons Dis.* **9**, S281–S295 (2019). 31498132 10.3233/JPD-191721 PMC6839496

[12] Horsager, J. et al. Brain-first versus body-first Parkinson’s disease: a multimodal imaging case-control study. *Brain* **143**, 3077–3088 (2020). 32830221 10.1093/brain/awaa238

[13] Borghammer, P. et al. Neuropathological evidence of body-first vs. brain-first Lewy body disease. *Neurobiol. Dis.* **161**, 105557 (2021). 34763110 10.1016/j.nbd.2021.105557

[14] Borghammer, P. et al. A postmortem study suggests a revision of the dual-hit hypothesis of Parkinson’s disease. *NPJ Parkinsons Dis.* **8**, 166 (2022). 36450732 10.1038/s41531-022-00436-2 PMC9712280

[15] Borghammer, P. The brain-first vs. body-first model of Parkinson’s disease with comparison to alternative models. *J. Neural Transm. (Vienna)* **130**, 737–753 (2023). 37062013 10.1007/s00702-023-02633-6

[16] Postuma, R. B. et al. MDS clinical diagnostic criteria for Parkinson’s disease. *Mov. Disord.* **30**, 1591–1601 (2015). 26474316 10.1002/mds.26424

[17] Shahnawaz, M. et al. Development of a biochemical diagnosis of Parkinson disease by detection of alpha-synuclein misfolded aggregates in cerebrospinal fluid. *JAMA Neurol.* **74**, 163–172 (2017). 27918765 10.1001/jamaneurol.2016.4547

[18] Groveman, B. R. et al. Rapid and ultra-sensitive quantitation of disease-associated alpha-synuclein seeds in brain and cerebrospinal fluid by alphaSyn RT-QuIC. *Acta Neuropathol. Commun.* **6**, 7 (2018). 29422107 10.1186/s40478-018-0508-2 PMC5806364

[19] Bongianni, M. et al. alpha-Synuclein RT-QuIC assay in cerebrospinal fluid of patients with dementia with Lewy bodies. *Ann. Clin. Transl. Neurol.* **6**, 2120–2126 (2019). 31599499 10.1002/acn3.50897 PMC6801172

[20] Manne, S. et al. Ultrasensitive detection of aggregated alpha-synuclein in glial cells, human cerebrospinal fluid, and brain tissue using the RT-QuIC assay: new high-throughput neuroimmune biomarker assay for Parkinsonian disorders. *J. Neuroimmune Pharm.* **14**, 423–435 (2019). 10.1007/s11481-019-09835-4 PMC6669119 30706414

[21] Shahnawaz, M. et al. Discriminating alpha-synuclein strains in Parkinson’s disease and multiple system atrophy. *Nature* **578**, 273–277 (2020). 32025029 10.1038/s41586-020-1984-7 PMC7066875

[22] Singer, W. et al. Alpha-synuclein oligomers and neurofilament light chain in spinal fluid differentiate multiple system atrophy from lewy body synucleinopathies. *Ann. Neurol.* **88**, 503–512 (2020). 32557811 10.1002/ana.25824 PMC7719613

[23] Bargar, C. et al. Streamlined alpha-synuclein RT-QuIC assay for various biospecimens in Parkinson’s disease and dementia with Lewy bodies. *Acta Neuropathol. Commun.* **9**, 62 (2021). 33827706 10.1186/s40478-021-01175-w PMC8028088

[24] Brockmann, K. et al. Association between CSF alpha-synuclein seeding activity and genetic status in Parkinson’s disease and dementia with Lewy bodies. *Acta Neuropathol. Commun.* **9**, 175 (2021). 34717775 10.1186/s40478-021-01276-6 PMC8556894

[25] Mammana, A. et al. RT-QuIC detection of pathological alpha-synuclein in skin punches of patients with Lewy body disease. *Mov. Disord.* **36**, 2173–2177 (2021). 34002890 10.1002/mds.28651 PMC8518528

[26] Orru, C. D. et al. A rapid alpha-synuclein seed assay of Parkinson’s disease CSF panel shows high diagnostic accuracy. *Ann. Clin. Transl. Neurol.* **8**, 374–384 (2021). 33373501 10.1002/acn3.51280 PMC7886040

[27] Perra, D. et al. Alpha-synuclein seeds in olfactory mucosa and cerebrospinal fluid of patients with dementia with Lewy bodies. *Brain Commun.* **3**, fcab045 (2021). 33870192 10.1093/braincomms/fcab045 PMC8042247

[28] Rossi, M. et al. Diagnostic value of the CSF alpha-synuclein real-time quaking-induced conversion assay at the prodromal MCI stage of dementia with Lewy bodies. *Neurology* **97**, e930–e940 (2021). 34210822 10.1212/WNL.0000000000012438 PMC8408510

[29] Russo, M. J. et al. High diagnostic performance of independent alpha-synuclein seed amplification assays for detection of early Parkinson’s disease. *Acta Neuropathol. Commun.* **9**, 179 (2021). 34742348 10.1186/s40478-021-01282-8 PMC8572469

[30] Sokratian, A. et al. Heterogeneity in alpha-synuclein fibril activity correlates to disease phenotypes in Lewy body dementia. *Acta Neuropathol.* **141**, 547–564 (2021). 33641009 10.1007/s00401-021-02288-1 PMC8055043

[31] Hall, S. et al. Performance of alphaSynuclein RT-QuIC in relation to neuropathological staging of Lewy body disease. *Acta Neuropathol. Commun.* **10**, 90 (2022). 35733234 10.1186/s40478-022-01388-7 PMC9219141

[32] Orimo, S. et al. Cardiac sympathetic denervation precedes neuronal loss in the sympathetic ganglia in Lewy body disease. *Acta Neuropathol.* **109**, 583–588 (2005). 15933869 10.1007/s00401-005-0995-7

[33] Matsubara, T. et al. Autopsy validation of the diagnostic accuracy of (123)I-metaiodobenzylguanidine myocardial scintigraphy for Lewy body disease. *Neurology* **98**, e1648–e1659 (2022). 35256483 10.1212/WNL.0000000000200110 PMC9052572

[34] Amino, T. et al. Profound cardiac sympathetic denervation occurs in Parkinson disease. *Brain Pathol.* **15**, 29–34 (2005). 15779234 10.1111/j.1750-3639.2005.tb00097.x PMC8095848

[35] Orimo, S., Suzuki, M., Inaba, A. & Mizusawa, H. 123I-MIBG myocardial scintigraphy for differentiating Parkinson’s disease from other neurodegenerative parkinsonism: a systematic review and meta-analysis. *Parkinsonism Relat. Disord.* **18**, 494–500 (2012). 22321865 10.1016/j.parkreldis.2012.01.009

[36] Nihashi, T., Ito, K. & Terasawa, T. Diagnostic accuracy of DAT-SPECT and MIBG scintigraphy for dementia with Lewy bodies: an updated systematic review and Bayesian latent class model meta-analysis. *Eur. J. Nucl. Med Mol. Imaging* **47**, 1984–1997 (2020). 31423561 10.1007/s00259-019-04480-8

[37] Tsujikawa, K. et al. Chronological changes of 123I-MIBG myocardial scintigraphy and clinical features of Parkinson’s disease. *J. Neurol. Neurosurg. Psychiatry* **86**, 945–951 (2015). 25935888 10.1136/jnnp-2015-310327

[38] Ryu, D. W. et al. Initial versus follow-up sequential myocardial 123I-MIBG scintigraphy to discriminate Parkinson disease from atypical Parkinsonian syndromes. *Clin. Nucl. Med* **44**, 282–288 (2019). 30589669 10.1097/RLU.0000000000002424

[39] Tanei, Z. I. et al. Lewy pathology of the esophagus correlates with the progression of Lewy body disease: a Japanese cohort study of autopsy cases. *Acta Neuropathol.* **141**, 25–37 (2021). 33150517 10.1007/s00401-020-02233-8 PMC7785549

[40] Samudra, N. et al. Clinicopathological correlation of cerebrospinal fluid alpha-synuclein seed amplification assay in a behavioral neurology autopsy cohort. *Alzheimers Dement*10.1002/alz.13799 (2024). 10.1002/alz.13799 PMC11095442 38539061

[41] Just, M. K. et al. Alpha-synuclein strain variability in body-first and brain-first synucleinopathies. *Front Aging Neurosci.* **14**, 907293 (2022). 35693346 10.3389/fnagi.2022.907293 PMC9178288

[42] Virameteekul, S., Revesz, T., Jaunmuktane, Z., Warner, T. T. & De Pablo-Fernandez, E. Clinical diagnostic accuracy of Parkinson’s disease: where do we stand? *Mov. Disord.* **38**, 558–566 (2023). 36602274 10.1002/mds.29317

[43] McKeith, I. G. et al. Diagnosis and management of dementia with Lewy bodies: fourth consensus report of the DLB Consortium. *Neurology* **89**, 88–100 (2017). 28592453 10.1212/WNL.0000000000004058 PMC5496518

[44] McKeith, I. G. et al. Research criteria for the diagnosis of prodromal dementia with Lewy bodies. *Neurology* **94**, 743–755 (2020). 32241955 10.1212/WNL.0000000000009323 PMC7274845

[45] Kurihara, M. et al. CSF P-Tau181 and other biomarkers in patients with neuronal intranuclear inclusion disease. *Neurology* **100**, e1009–e1019 (2023). 36517236 10.1212/WNL.0000000000201647 PMC9990848

[46] Kurihara, M. et al. Neuropathological changes associated with aberrant cerebrospinal fluid p-tau181 and Abeta42 in Alzheimer’s disease and other neurodegenerative diseases. *Acta Neuropathol. Commun.* **12**, 48 (2024). 38539238 10.1186/s40478-024-01758-3 PMC10976730

[47] Kurihara, M. et al. Relationship Between Cerebrospinal Fluid Alzheimer’s Disease Biomarker Values Measured via Lumipulse Assays and Conventional ELISA: Single-Center Experience and Systematic Review. *J. Alzheimers. Dis.* **99**, 1077–1092 (2024). 38759016 10.3233/JAD-240185 PMC11191528

[48] Goto, R. et al. Correlations between cerebrospinal fluid homovanillic acid and dopamine transporter SPECT in degenerative parkinsonian syndromes. *J. Neural Transm. (Vienna)* **130**, 513–520 (2023). 36871130 10.1007/s00702-023-02611-y PMC10050014

[49] Shimasaki, R. et al. Associations of cerebrospinal fluid monoamine metabolites with striatal dopamine transporter binding and ^123^I-meta-iodobenzylguanidine cardiac scintigraphy in Parkinson's disease: Multivariate analyses. *Parkinsonism Relat. Disord.* **128**, 107129 (2024). 39241507 10.1016/j.parkreldis.2024.107129

[50] Tossici-Bolt, L. et al. [(123)I]FP-CIT ENC-DAT normal database: the impact of the reconstruction and quantification methods. *EJNMMI Phys.* **4**, 8 (2017). 28130765 10.1186/s40658-017-0175-6 PMC5272851

[51] Matsuda, H. et al. Japanese multicenter database of healthy controls for [(123)I]FP-CIT SPECT. *Eur. J. Nucl. Med Mol. Imaging* **45**, 1405–1416 (2018). 29478082 10.1007/s00259-018-3976-5 PMC5993845

[52] Nakajima, K. et al. Multicenter cross-calibration of I-123 metaiodobenzylguanidine heart-to-mediastinum ratios to overcome camera-collimator variations. *J. Nucl. Cardiol.* **21**, 970–978 (2014). 24942608 10.1007/s12350-014-9916-2 PMC4167440

[53] Kanda, Y. Investigation of the freely available easy-to-use software ‘EZR’ for medical statistics. *Bone Marrow Transpl.* **48**, 452–458 (2013). 10.1038/bmt.2012.244 PMC3590441 23208313

