# Supplementary material for: α-synuclein seed amplification assay sensitivity may be associated with cardiac MIBG abnormality among patients with Lewy body disease
Source: NPJ Parkinsons Dis. 2024 Oct 21;10:190. doi: 10.1038/s41531-024-00806-y (PMC11494045; doi:10.1038/s41531-024-00806-y)
Supplement: Supplementary file 1 — Supplementary Material [file 41531_2024_806_MOESM1_ESM.docx]

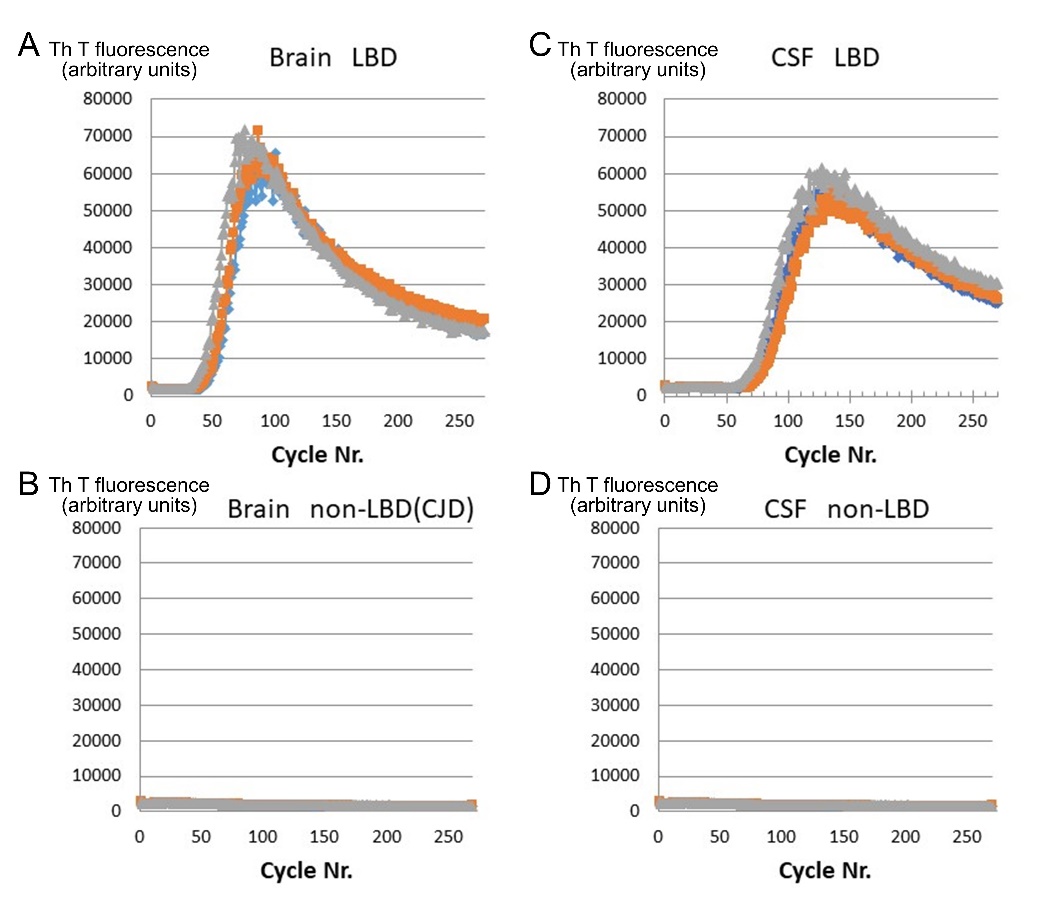


**Supplementary Figure 1. Preliminary experiments of α-syn RT-QuIC assay used in this study.**

Preliminary experiments of α-syn RT-QuIC assay used in this study using brain homogenates or CSF samples of LBD and CJD. Different colors indicate three technical replicates. (A, B) Brain homogenates of LBD consistently increased ThT fluorescence while those of CJD did not as previously described. (C, D) Similarly, CSF of LBD consistently increased ThT fluorescence while those of CJD did not, confirming the specificity of the α-syn RT-QuIC assay used in this study.

Abbreviations: CJD, Creutzfeldt–Jakob disease; CSF, cerebrospinal fluid; LBD, Lewy body disease; Nr, number; ThT, thioflavin T


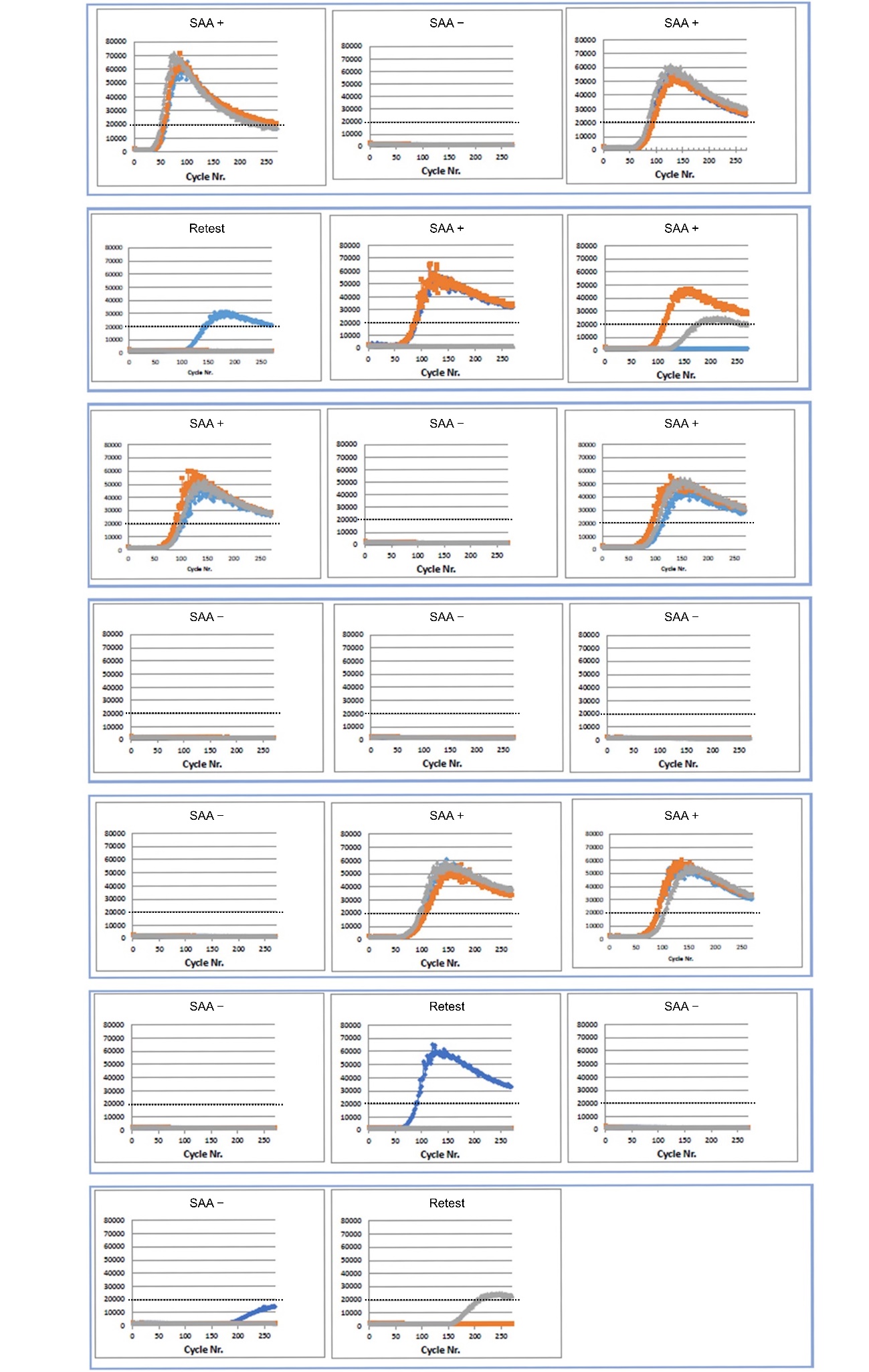
 **Supplementary Figure 2. Example α-syn SAA curves and interpretations**

Vertical axes show thioflavin T (ThT) fluorescence (arbitrary units) and different colors indicate three technical replicates for the same CSF sample. Horizontal dotted lines show the cut-off value of 20,000 arbitrary units. The interpretation of the first analyses for each CSF sample is provided as an example. Criteria for determining a positive reaction was as follows. 1. A positive reaction is indicated when the ThT value exceeds 20,000 arbitrary units in two or more wells within 200 cycles. 2. In instances where only a single well of the sample exhibits an elevated value, the sample was retested. 3. Subsequently, a positive reaction is confirmed if more than one well demonstrates a ThT value of 20,000 arbitrary units or greater upon retesting.

Abbreviations: Nr, number; SAA, seed amplification assay; +, positive; −, negative

|  | α-syn SAA positive | α-syn SAA negative | p value | |
| --- | --- | --- | --- | --- |
| n = | 25 | 16 |  |  |
| Age (years) | 73.5 ±9.1 | 74.4 ±9.1 | 0.75^a^ |  |
| Sex (female) | 44.0% | 43.8% | 1^b^ |  |
| Disease duration (years) | 1 [1–2.0] | 1 [1–1.25] | 0.51^c^ |  |
| UPDRS Part 3 score | 29.9±15.5 | 21.8±13.5 | 0.10^a^ |  |
| DAT Z-SBR | -3.52 ±1.02 | -3.21 ±0.86 | 0.32^a^ |  |
| DLB | 24.0% | 6.2% | 0.215^b^ |  |
| LP to SAA (days) | 334 ±87 | 380 ±86 | 0.105^a^ |  |
| MIBG abnormal | 92% | 25% | < 0.001^b^ | *** |
| early H/M ratio | 1.67 ±0.41 | 2.66 ±0.37 | < 0.001^a^ | *** |
| delayed H/M ratio | 1.46 ±0.47 | 2.67 ±0.52 | < 0.001^a^ | *** |
| wash out rate | 57.4 ±21.9% | 23.0 ±16.9 | < 0.001^a^ | *** |

**Supplementary Table 1. Baseline characteristics of participants with LBD grouped by α-syn SAA positivity**

P-values represent the result of a, Student’s t-test; b, Fisher’s exact test; c, Mann–Whitney U test

Abbreviations: DAT, dopamine transporter; DLB, dementia with Lewy bodies; H/M, heart-to-mediastinum; LP, lumbar puncture; SAA, seed amplification assay; UPDRS, unified Parkinson's disease rating scale; Z-SBR, Z-score of the average striatal SBR


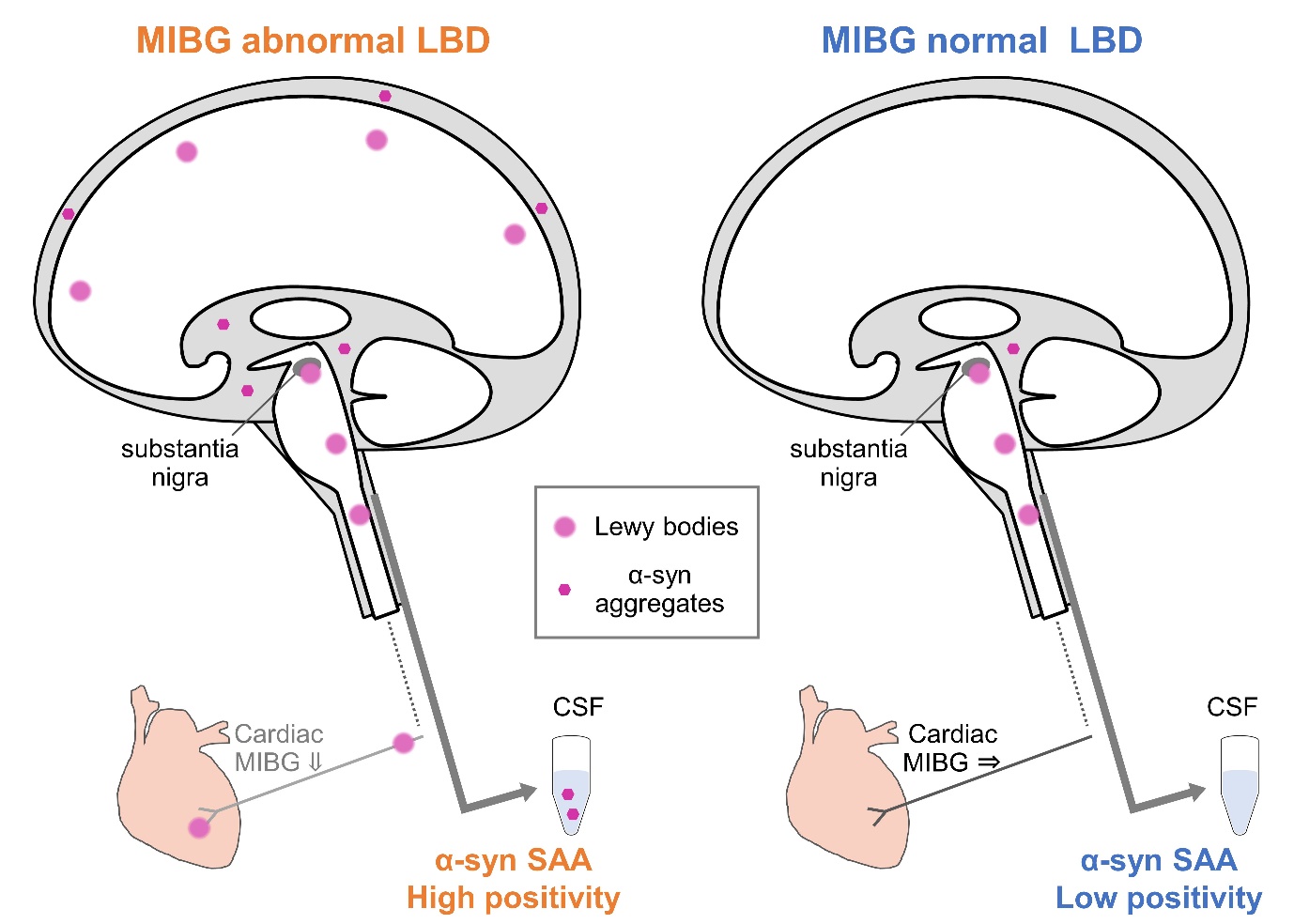


**Supplementary Figure 3. Schematic illustration of the hypothesis involving different α-syn distribution and load contributing to the difference in SAA positivity between MIBG abnormal and normal group**


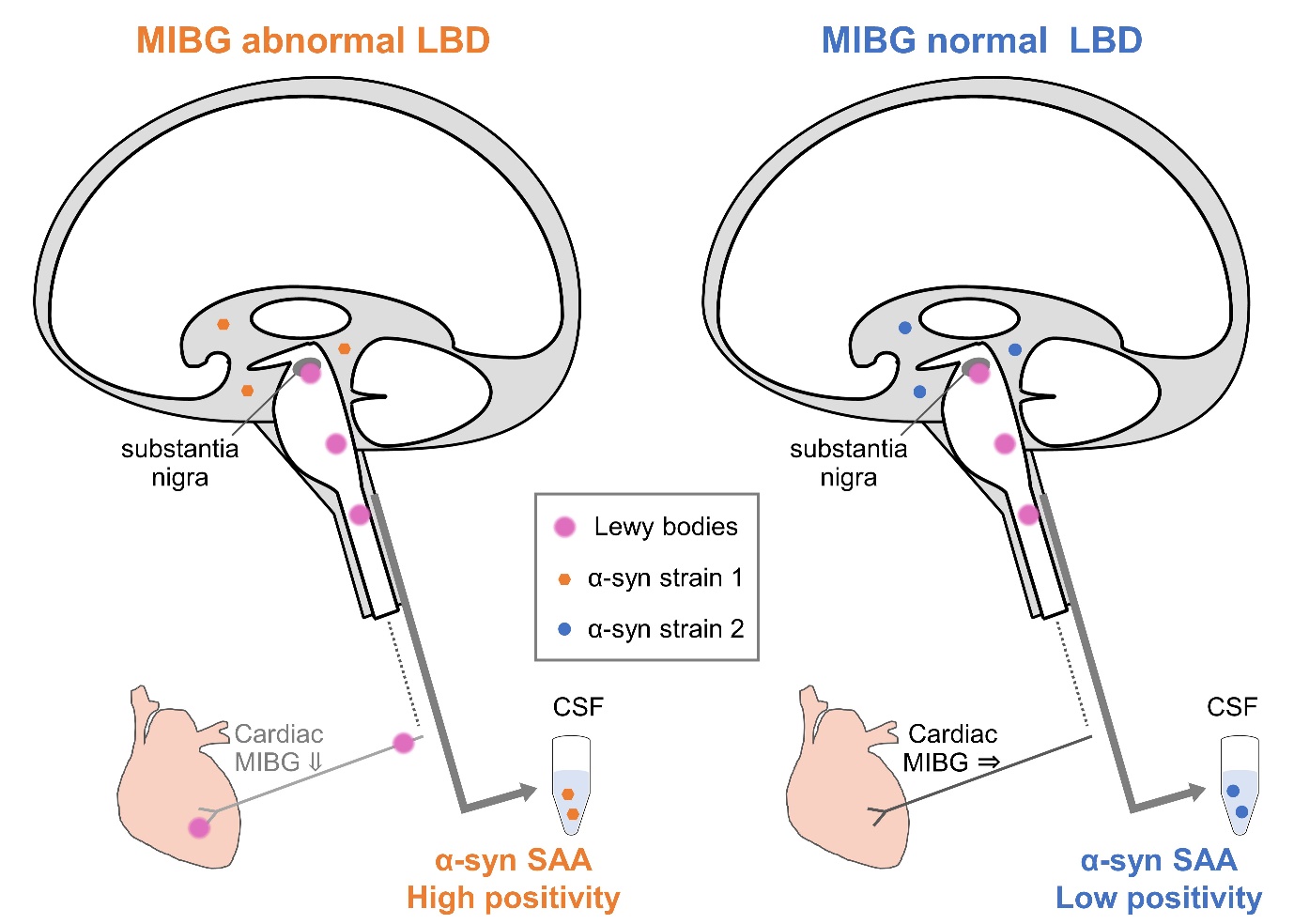


**Supplementary Figure 4. Schematic illustration of the hypothesis involving different α-syn strains contributing to the difference in SAA positivity between MIBG abnormal and normal group**
